# Supplementary material for: Crbn modulates calcium influx by regulating Orai1 during efferocytosis
Source: Nat Commun. 2020 Oct 30;11:5489. doi: 10.1038/s41467-020-19272-0 (PMC7603501; doi:10.1038/s41467-020-19272-0)
Supplement: Supplementary file 1 — Supplementary Information [file 41467_2020_19272_MOESM1_ESM.pdf]

Supplementary Figures

Crbn modulates calcium influx by regulating Orai1 during efferocytosis

Hyunji Moon[1, 2], Chanhyuk Min[1, 2], Gayoung Kim[1], Deokhwan Kim[1, 2], Kwanhyeong Kim[1, 2], Sang-Ah Lee[1, 2], Byeongjin Moon[1, 2], Susumin Yang[1, 2], Juyeon Lee[1, 2], Seung-Joo Yang[1], Steve K. Cho[1], Gwangrog Lee[1, 2], Chang Sup Lee[3], Chul-Seung Park[1], Daeho Park[1, 2, 4, *]

1. School of Life Sciences, Gwangju Institute of Science and Technology, Gwangju 61005, Korea

2. Cell Mechanobiology Laboratory, Gwangju Institute of Science and Technology, Gwangju 61005, Korea

3. College of Pharmacy and Research Institute of Pharmaceutical Sciences, Gyeongsang National University, Jinju 52828, Korea

4. Research Center for Cellular Homeostasis, Ewha Womans University, Seoul 03760, Korea

*** Correspondence to**

Daeho Park

School of Life Sciences

Gwangju Institute of Science and Technology

Gwangju 61005, Korea

Tel.: 82-62-715-2890

Fax: 82-62-715-2484

E-mail: daehopark@gist.ac.kr

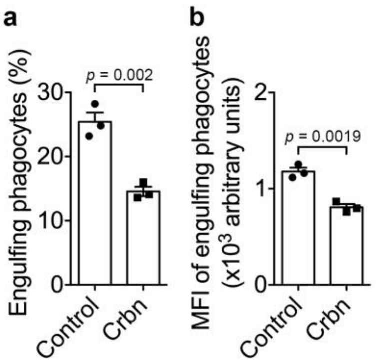

Supplementary Fig. 1: Crbn overexpression decreases efferocytosis

a, **b** MEFs transfected with HA-Crbn were incubated with TAMRA-stained apoptotic thymocytes for 2 h. After that, the cells were incubated with an anti-HA antibody, labelled with an Alexa 488-conjugated secondary antibody, and analyzed using flow cytometry. HA- and TAMRA-positive cells were considered as Crbn expressing phagocytes engulfing apoptotic cells. n=3 experiments. Mean ± SEM (Two-tailed unpaired Student t test).

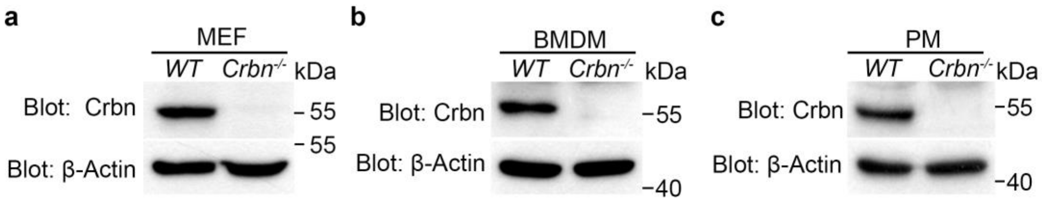

Supplementary Fig. 2: Expression of Crbn in phagocytes derived from *Crbn*$^{-/-}$ mice

a-**c** MEFs (**a**), BMDMs (**b**), or peritoneal macrophages (**c**) derived from *WT* or *Crbn*$^{-/-}$ mice were lysed, and Crbn in the lysates was detected with an anti-Crbn antibody. Images are representative of at least three independent experiments. PM, peritoneal macrophage. Data are representative of three (**a**, **b**, **c**) independent experiments.

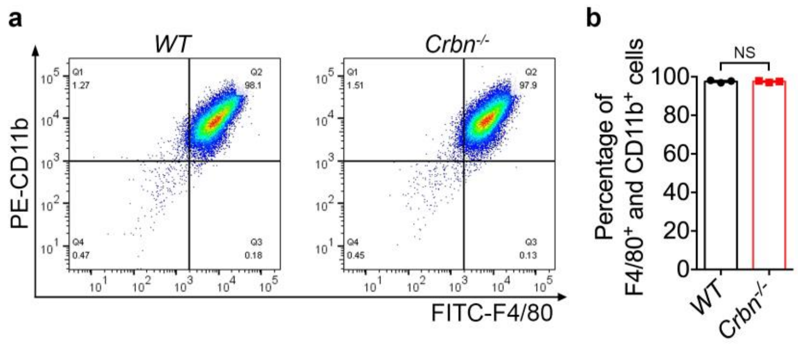

Supplementary Fig. 3: BMDMs derived from *WT* and *Crbn*$^{-/-}$ mice show a similar degree of differentiation

a, **b** BMDMs derived from *WT* and *Crbn*$^{-/-}$ mice were stained with PE-conjugated anti-CD11b and FITC-conjugated F4/80 antibodies and analyzed by flow cytometry. Representative dot plots were shown (**a**) and CD11b- and F4/80-positive cells were quantified (**b**). n=3 experiments. Mean ± SEM. NS, not significant (Two-tailed unpaired Student t test).

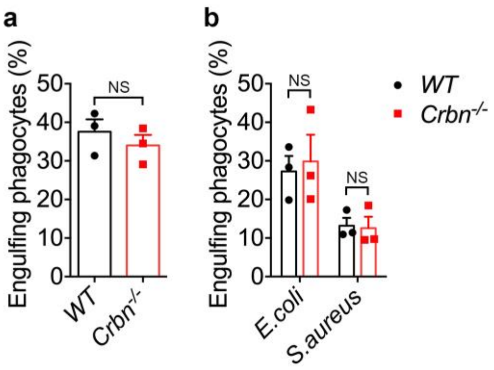

Supplementary Fig. 4: Crbn does not affect phagocytosis of non-apoptotic targets

a, **b** MEFs derived from *WT* and *Crbn*$^{-/-}$ mice incubated with polystyrene beads for 1 h (**a**) or bioparticles (*E. coli* and *S. aureus* particles) for 2 h (**b**) and analyzed by flow cytometry. n=3 experiments. Mean ± SEM. NS, not significant (Two-tailed unpaired Student t test).

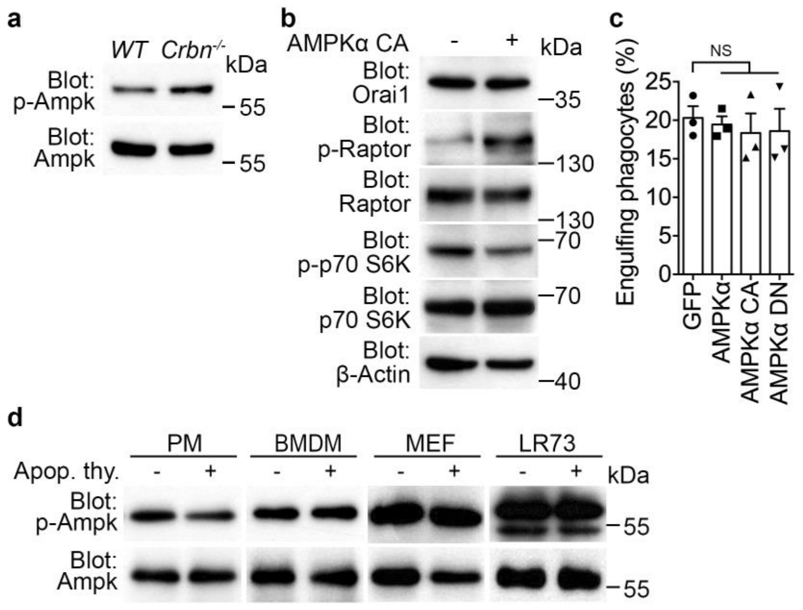

Supplementary Fig. 5: Ampk does not promote efferocytosis

a BMDMs derived from *WT* or *Crbn*$^{-/-}$ mice were lysed and the levels of phosphorylated Ampk in the lysates were detected with an anti-phospho-Ampk antibody. Data are representative of four independent experiments. **b** LR73 cells were transfected with a constitutive active form of Ampk. 1 d after transfection, the cells were lysed and proteins in the lysates were detected with the indicated antibodies. Data are representative of three independent experiments. **c** LR73 cells were transfected with the indicated plasmids. At 1 day after transfection, the cells were incubated with TAMRA-stained apoptotic thymocytes for 2 h, washed with PBS, trypsinized, and analyzed by flow cytometry. n=3 experiments. Mean ± SEM. NS, not significant (One-way ANOVA). **d** The indicated phagocytes were incubated with apoptotic thymocytes and then Ampk phosphorylation was detected with an anti-phospho-Ampk antibody. Data are representative of three independent experiments. PM, peritoneal macrophage. NS, not significant.

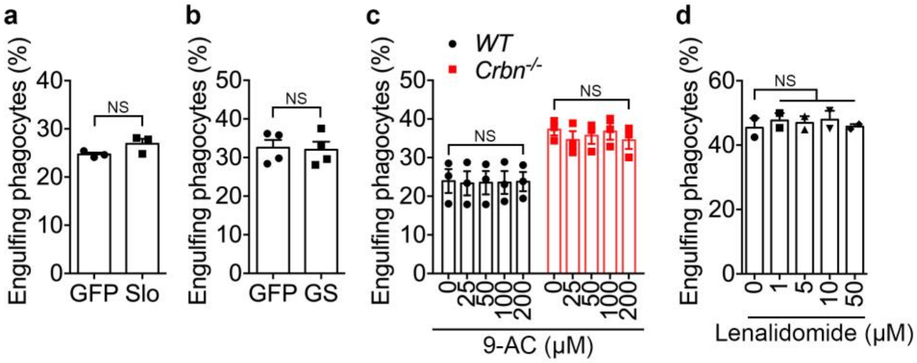

Supplementary Fig. 6: Known Crbn substrates are not involved in efferocytosis modulated by Crbn

a, **b** LR73 cells were transfected with the indicated plasmids. At 1 day after transfection, the cells were incubated with TAMRA-stained apoptotic thymocytes for 2 h. Engulfing phagocytes were analyzed by flow cytometry. n=3 (**a**), n=4 (**b**) experiments. Mean ± SEM. NS, not significant (Two-tailed unpaired Student t test). **c** BMDMs derived from *WT* or *Crbn*$^{-/-}$ mice were incubated with TAMRA-stained apoptotic thymocytes in the presence of the indicated concentrations of 9-AC for 2 h. Thereafter, the cells were analyzed by flow cytometry. n=3 experiments. Mean ± SEM. NS, not significant (One-way ANOVA). **d** THP-1 derived macrophages were pre-treated with the indicated concentrations of lenalidomide for 24 h. Thereafter, the cells were incubated with TAMRA-stained apoptotic thymocytes for 2 h in the presence of lenalidomide. Engulfing phagocytes were analyzed by flow cytometry. n=3 experiments. Mean ± SEM. NS, not significant (One-way ANOVA).

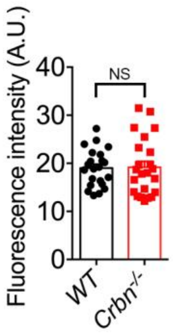

Supplementary Fig. 7: The total intensity of phalloidin around PS beads

BMDMs derived from *WT* or *Crbn*$^{-/-}$ mice were incubated with PS beads, stained with phalloidin, and observed by confocal microscopy. The intensity of phalloidin around the targets was quantified using ImageJ. Data are shown as the mean ± standard error of mean. NS, not significant. n=22 PS beads for *WT* and n=23 PS beads for *Crbn*$^{-/-}$. Mean ± SEM. NS, not significant (Two-tailed unpaired Student t test).

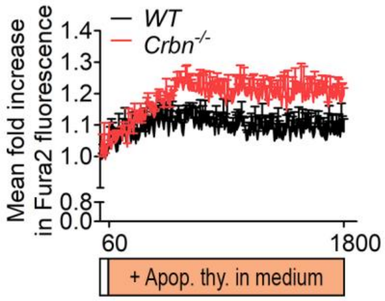

Supplementary Fig. 8: Intracellular calcium measurement during using Fura2-AM during efferocytosis

BMDMs from the indicated mice were stained with Fura2-AM, and then apoptotic cells in RPMI were added at the indicated time. Fluorescence of BMDMs was measured with a microplate reader (FlexStation 3). n=3 experiments. Mean ± SEM.

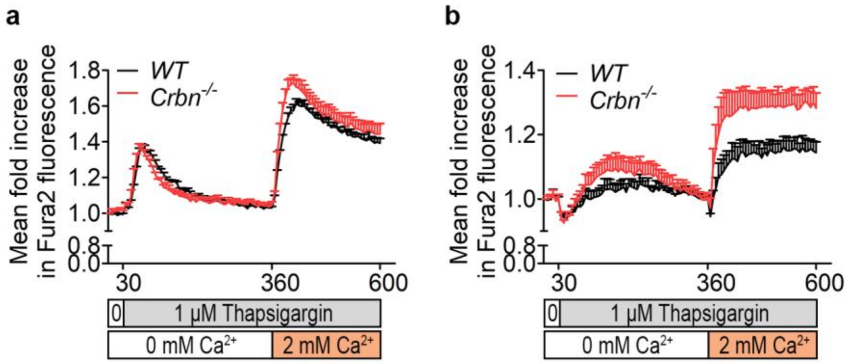

Supplementary Fig 9. SOCE increases in phagocytes derived from *Crbn*$^{-/-}$ mice

a, **b** Peritoneal macrophages (**a**) or MEFs (**b**) derived from *WT* or *Crbn*$^{-/-}$ mice were stained with Fura2-AM, and then the indicated concentration of thapsigargin and 2 mM calcium were sequentially added to the cells at the indicated time. The fluorescence of phagocytes was measured with a microplate reader. n=3 experiments. Mean ± SEM.

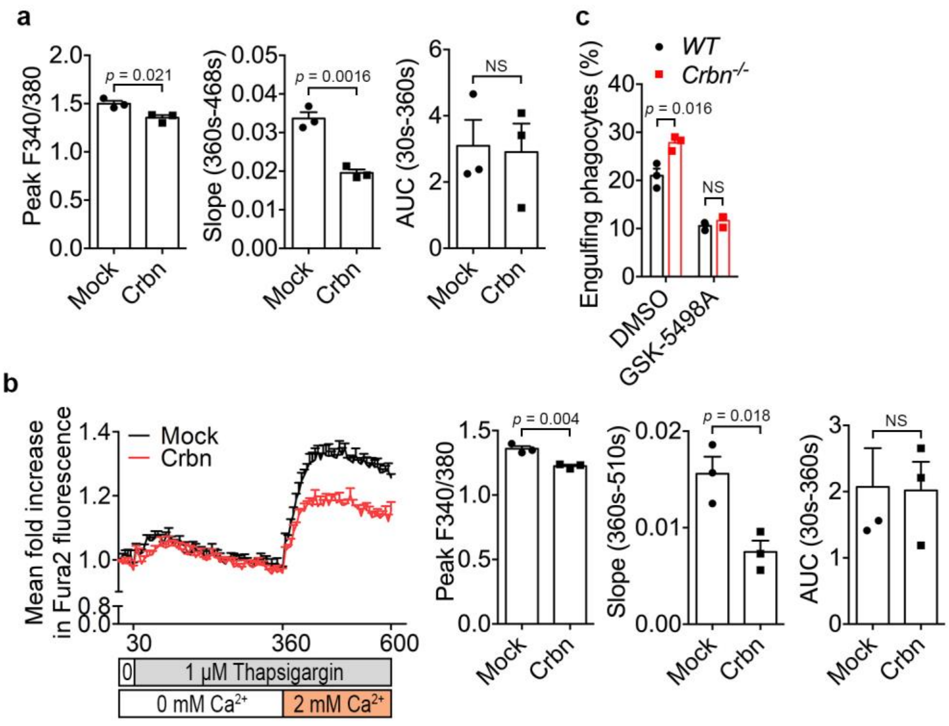

Supplementary Fig. 10: Crbn overexpression decreases SOCE

a, **b** Crbn-overexpressing LR73 (**a**) cells or MEFs (**b**) were stained with Fura2-AM and then treated with 1 µM thapsigargin for the indicated duration. Thereafter, 2 mM calcium was added to the cells at the indicated time. Fluorescence of the cells was measured with a microplate reader, and the peak, slope, and AUC of the graph was calculated. The graph for Crbn-overexpressing LR73 cells is shown in **Fig. 3b**. AUC, area under curve. n=3 experiments. Mean ± SEM. NS, not significant (Two-tailed unpaired Student t test). **c** BMDMs derived from *WT* or *Crbn*$^{-/-}$ mice were incubated with TAMRA-stained apoptotic cells in GSK-5498A (50 µM). Thereafter, engulfing phagocytes were analyzed by flow cytometry. n=3 experiments. Mean ± SEM. NS, not significant (Two-tailed unpaired Student t test).

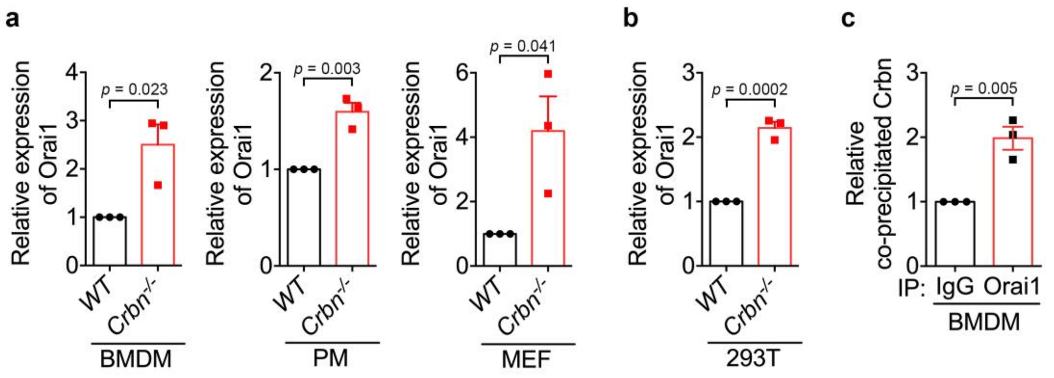

Supplementary Fig. 11: Quantification of immunoblots

a The levels of Orai1 in BMDM, PM, and MEF derived from *WT* and *Crbn*$^{-/-}$ mice were quantified in **Fig. 4a**. n=3 experiments. **b** The levels of Orai1 in *WT* and *Crbn*$^{-/-}$ 293T cells were quantified in **Fig. 4b**. n=3 experiments. **c** BMDMs were lysed and then the lysates were incubated with an anti-Orai1 antibody or a control antibody and protein A/G conjugated agarose beads. Bead-bound proteins were detected with the indicated antibodies and quantified in **Fig. 4f**. n=3 experiments. For all panels: mean ± SEM (Two-tailed unpaired Student t test).

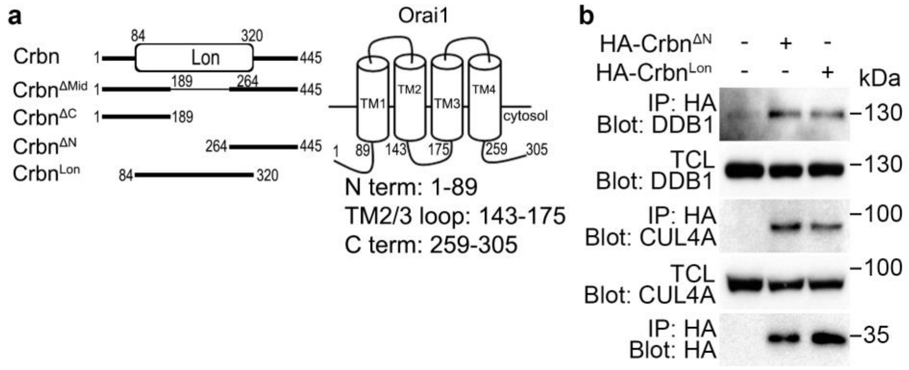

Supplementary Fig. 12: Crbn and Orai1 constructs used in the study and interaction of Crbn fragments with the components of the CRL4^{CRBN} E3 ubiquitin ligase

a Schematic diagram of Crbn and Orai1 constructs. **b** 293T cells transfected with the indicated plasmids were lysed, and the lysates were incubated with an anti-HA antibody and protein A/G conjugated agarose beads. Bead-bound proteins and proteins in the lysates were detected with the indicated plasmids. Data are representative of three independent experiments.

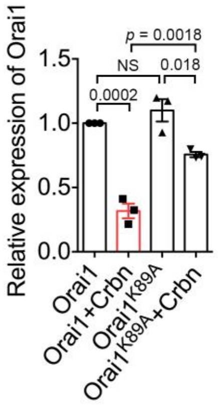

Supplementary Fig. 13: Quantification of immunoblots in Fig. 6c

The levels of Orai1 and Orai1^{K89A} in **Fig. 6c** were quantified. n=3 experiments. Mean ± SEM. The numbers in the graph indicate *p* values. NS, not significant (Two-tailed unpaired Student t test).

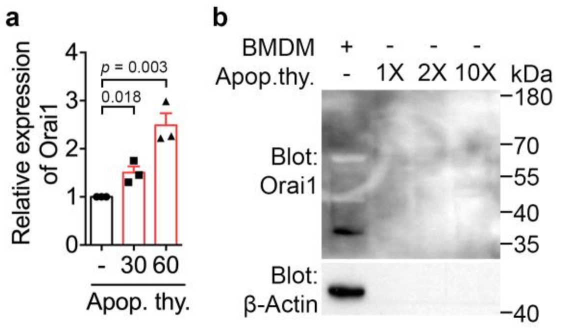

Supplementary Fig. 14: Quantification of immunoblots in Fig. 6d and the levels of Orai1 in apoptotic thymocytes

a BMDMs were incubated with apoptotic thymocytes for the indicated times and then lysed. Orai1 in the lysates was detected using immunoblotting and quantified. n=3 experiments. Mean ± SEM. The numbers in the graph indicate *p* values (One-way ANOVA). **b** BMDMs and apoptotic thymocytes were lysed, and Orai1 in the lysates was detected. The number of apoptotic cells used in the experiment was 1, 2, and 10 times higher than in **Fig. 6d**. In addition, the actual number of apoptotic cells after PBS washing in **Fig. 6d** might be much less than the number of apoptotic cells (1X) added to phagocytes.

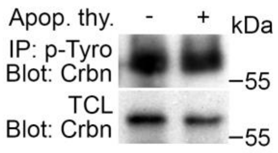

Supplementary Fig. 15: Tyrosine phosphorylation of Crbn during efferocytosis
LR73 cells were incubated with apoptotic cells and lysed. The lysates were incubated with an anti-phospho-tyrosine antibody and protein A/G conjugated agarose beads. Co-precipitated Crbn was detected. Images are representative of two independent experiments.

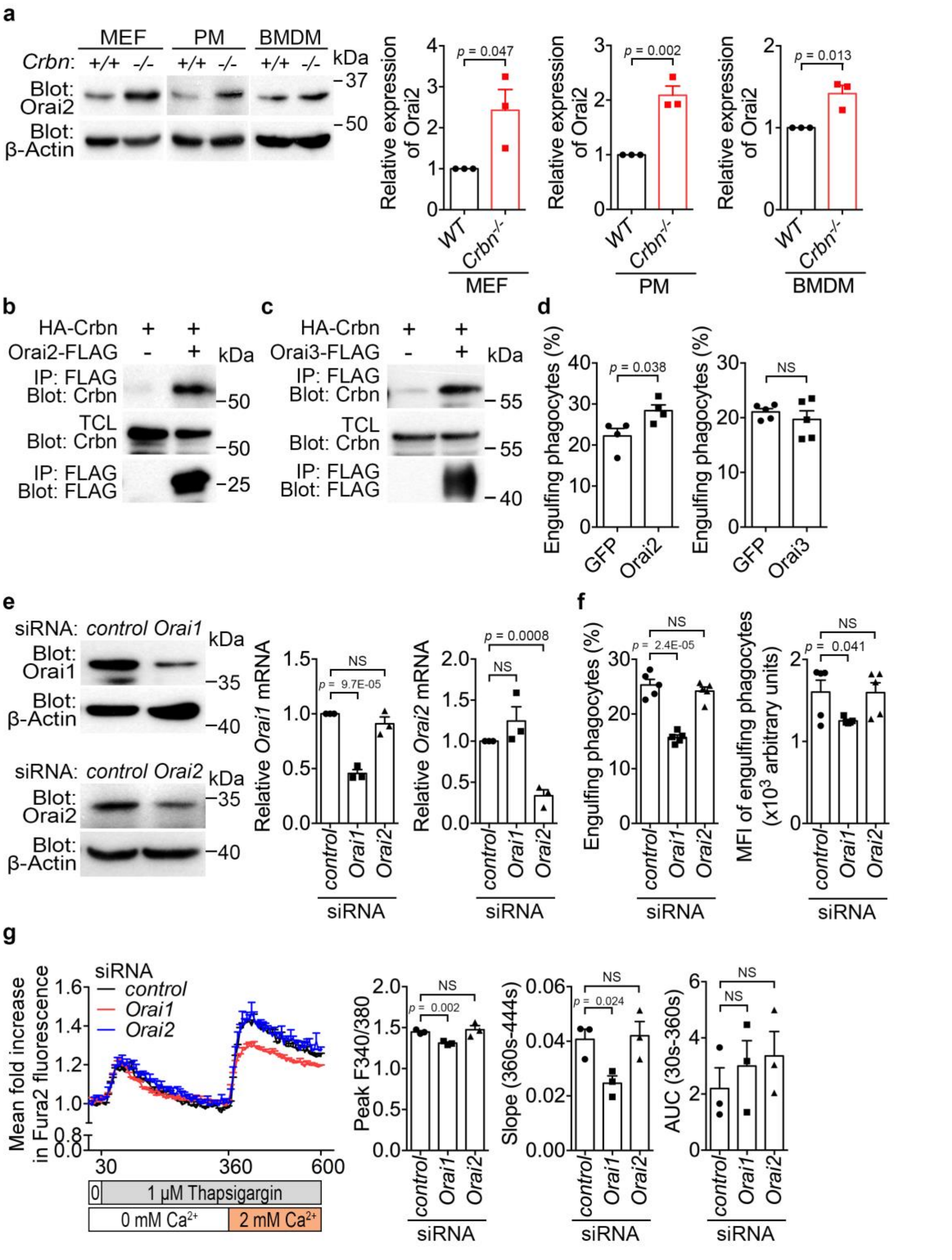

Supplementary Fig. 16: The effects of Orai1 homologs on efferocytosis and SOCE

a Phagocytes derived from the indicated mice were lysed and the levels of Orai2 in the lysates were detected with an anti-Orai2 antibody and quantified. n=3 experiments.

Mean ± SEM (Two-tailed unpaired Student t test). **b**, **c** 293T cells were transfected with the indicated plasmids. At 2 days after transfection, the cells were lysed and Orai2-FLAG (**b**) or Orai3-FLAG (**c**) in the lysates was precipitated with an anti-FLAG antibody. Proteins in the precipitants were detected with the indicated antibodies. **d** LR73 cells transfected with the indicated plasmids were incubated with TAMRA-stained apoptotic thymocytes for 2 h, washed with PBS, trypsinized, and analyzed by flow cytometry. n=4 (for Orai2), n=5 (for Orai3) experiments. Mean ± SEM. NS, not significant (Two-tailed unpaired Student t test). **e** MEFs were transfected with *control*, *Orai1*, or *Orai2* siRNA. 1 d after transfection, the cells were lysed, and then Orai1 and Orai2 in the lysates were detected using immunoblotting (left). The transcript levels of *Orai1* and *Orai2* in MEFs transfected with *control*, *Orai1*, or *Orai2* siRNA were also measured using quantitative RT-PCR (right). n=3 experiments. Mean ± SEM NS, not significant (One-way ANOVA). **f** MEFs were transfected with *control*, *Orai1*, or *Orai2* siRNA. 1 d after transfection, the cells were incubated with TAMRA-stained apoptotic thymocytes for 2 h and then analyzed by flow cytometry. n=5 experiments. Mean ± SEM. NS, not significant (One-way ANOVA). **g** MEFs transfected with the indicated siRNA were stained with Fura2-AM and then treated with 1 μM thapsigargin for the indicated duration. Thereafter, 2 mM calcium was added to the cells at the indicated time. Fluorescence of the cells was measured with a microplate reader. n=3 experiments. Mean ± SEM. NS, not significant (One-way ANOVA).

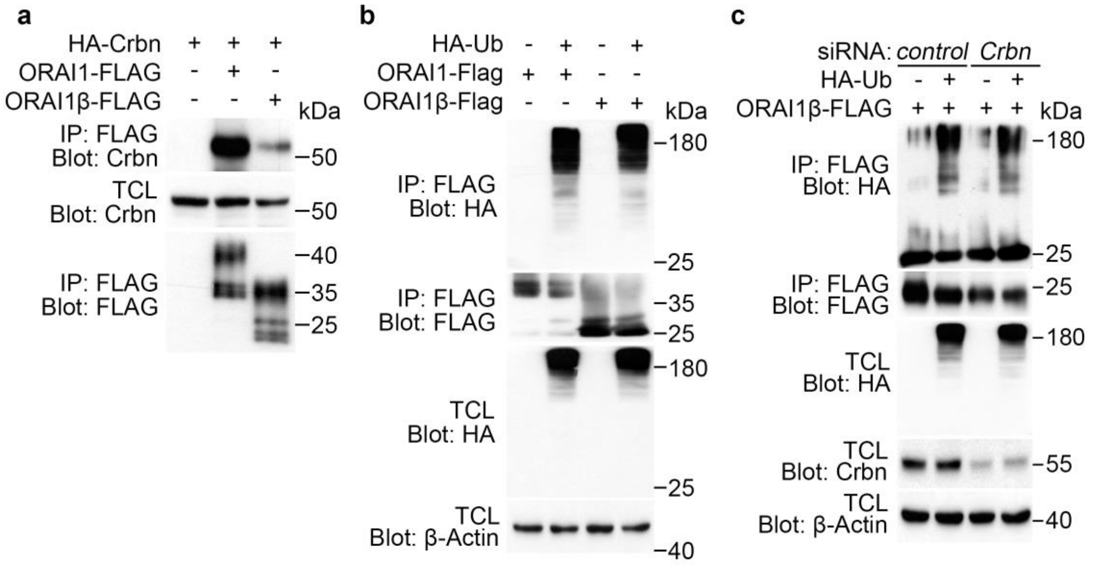

Supplementary Fig. 17: Interaction of Crbn with ORAI1β and Ubiquitination of ORAI1β

a, **b** 293T cells transfected with the indicated plasmids were lysed, and the lysates were incubated with an anti-FLAG antibody-conjugate agarose beads. Bead-bound proteins were detected with the indicated antibodies. Images are representative of two independent experiments.

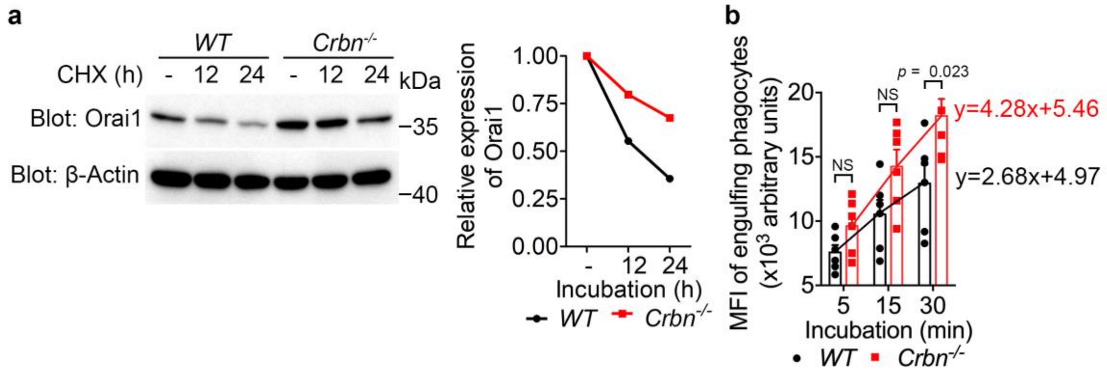

Supplementary Fig. 18: *Crbn$^{-/-}$* phagocytes are more efficient to continuously engulf apoptotic cells

a *WT* and *Crbn$^{-/-}$* 293T cells were incubated with cycloheximide for the indicated times and then lysed. Orai1 in the lysates was detected using immunoblotting. **b** BMDMs derived from *WT* or *Crbn$^{-/-}$* mice were incubated with TAMRA-stained apoptotic thymocytes for the indicated times and engulfing phagocytes were analyzed by flow cytometry. n=6 experiments. Mean ± SEM. NS, not significant (Two-tailed unpaired Student t test).

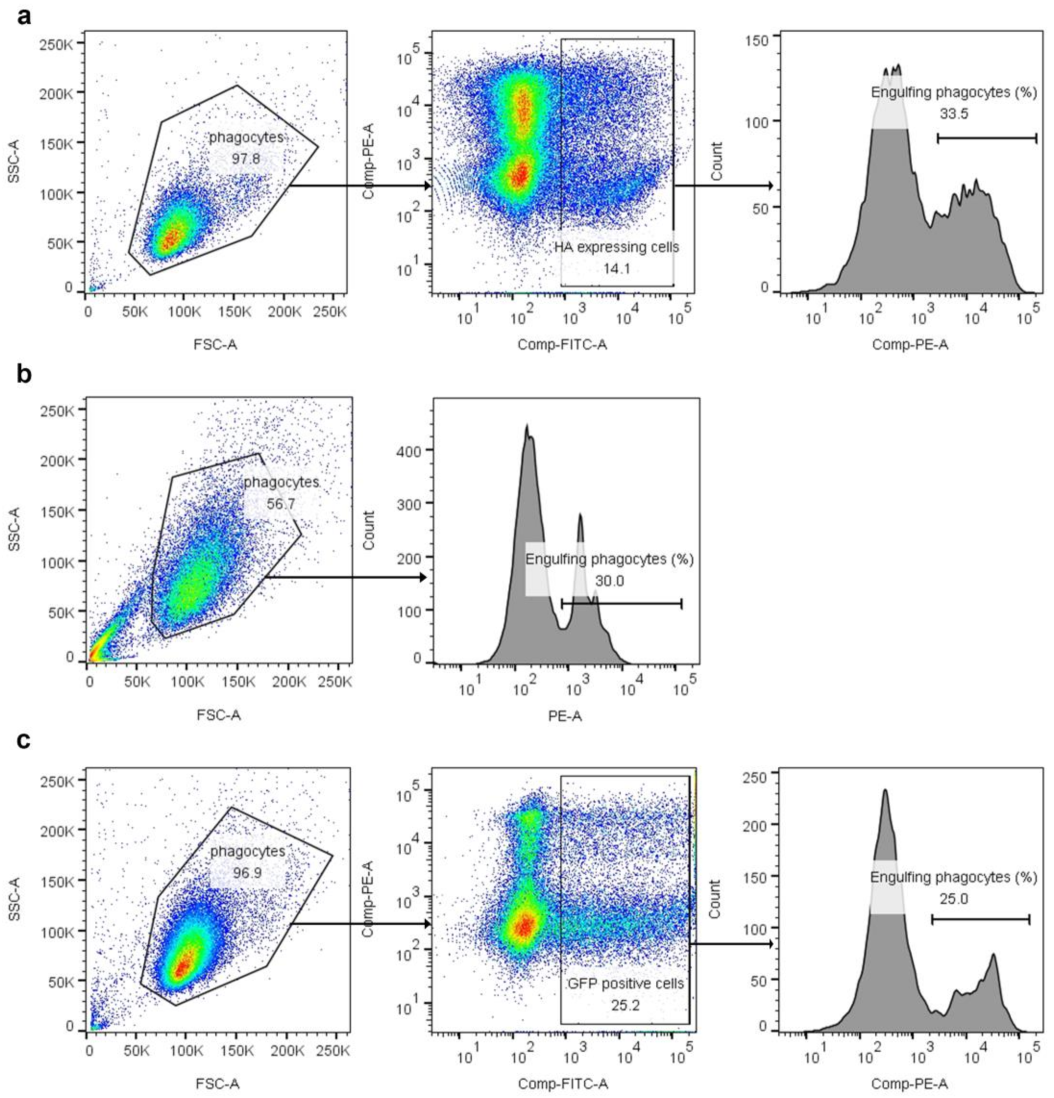

Supplementary Fig. 19: Gating strategies to determine the percentage of phagocytes engulfing apoptotic cells.

a Phagocytes transfected with Crbn were incubated with TAMRA-stained apoptotic cells and stained by an anti-HA (Crbn) antibody. Phagocytes were gated based on SSC and FSC to distinguish them from non-engulfed apoptotic cells and then double positive cells for HA (FITC) and TAMRA (PE) were considered as engulfing phagocytes. This gating strategy was used for Fig.1a, b; Fig. 6a, b; Supplementary fig. 1a, b. **b** Phagocytes were separated from non-engulfing apoptotic cells using gating based on FSC and SSC and then TAMAR (PE) positive cells were considered as phagocytes engulfing apoptotic cells. This gating strategy was used for Fig. 1c, d; 3d, e, h; Supplementary fig. 4a, 4b,

6c, 6d, 10c, 16f, 18b. **c** Phagocytes were transfected by the indicated plasmids with GFP and incubated with apoptotic cells. The same gating strategy was used as in (**a**). Double positive cells for GFP (transfected cells) and TAMRA (PE) were considered as phagocytes engulfing apoptotic cells. This gating strategy (**c**) was used for Supplementary fig. 5c, 6a, 6b, 16d.

Supplementary Table

Primers used in this study

Gene	Purpose		Primer sequence (5' to 3')
Mouse Orai1	RT-PCR<br>Q-RT-PCR	Forward	CAA GCT CTA CTT AAG CCG CGC C
		Reverse	CGA TGT TGG GCA GGA TGC AGG T
Mouse Orai2	RT-PCR<br>Q-RT-PCR	Forward	CCT GGA AGC TCT ACC 557 TGA G
		Reverse	GCT GAT GAG CAG AGC AAA CAG ATG
Mouse Orai3	RT-PCR<br>Q-RT-PCR	Forward	ACC GCG 558 GCT ACC TGG ACC TTA
		Reverse	GCA GGC CTA GTG GGT ATT CAT
Mouse β-Actin	RT-PCR<br>Q-RT-PCR	Forward	TCT GGC ACC ACA CCT TCT ACA AT
		Reverse	GAT CTG GGT CAT CTT TTC ACG GTT
Mouse Orai1	Cloning<br>Into pEBB-Flag	Forward	ATT GCT AGC ATG AGC CTC AAC GAG CAC TCG
		Reverse	ATT GGT ACC GGC ATA GTG GGT GCC CGG
Mouse Orai2	Cloning<br>Into pEBB-Flag	Forward	ATT ACT AGT ATG AGT GCA GAG CTC AAT GTG
		Reverse	ATT GTT AAC CAC CAC CTG CAG GCT CCG
Mouse Orai3	Cloning<br>Into pEBB-Flag	Forward	ATT GCT AGC ATG AAG GGC GGC GAG GGG
		Reverse	ATT GTC GAC CAC AGC CTG CAG CTC CCC
Crbn	Cloning into<br>pGBT10 vector	Forward	ATT ACT AGT ATG GCC GGC GAA GGA GAT CAC
		Reverse	ATT GCG GCC GCT TAT AAG CAA AGT ATT AC
Orai1 N term	Cloning into<br>pVP16 vector	Forward	ATT ACT AGT ATG AGC CTC AAC GAG CAC TCG ATG
		Reverse	ATT GGT ACC TTA TTT GAG CTT GGC GCG GCT TAA GTA
Orai1 N term	Cloning into<br>pEBG-GST vector	Forward	ATT ACT AGT ATG AGC CTC AAC GAG CAC TCG ATG
		Reverse	ATT GGT ACC TTA TTT GAG CTT GGC GCG GCT TAA GTA
Orai1 Loop	Cloning into<br>pEBG-GST vector	Forward	ATT ACT AGT ATG AGC ACC TGC ATC CTG CCC
		Reverse	ATT GGT ACC TTA CTC GAT GTG GCG ATG CAT

Orai1 C term	Cloning into pEBG-GST vector	Forward	ATT ACT AGT ATG CAC TTC TAC CGC TCC CTG GTC
		Reverse	ATT GGT ACC TTA GGC ATA GTG GGT GCC CGG
Crbn-△Mid	Cloning into pcDNA3.0-HA vector	Forward	FULL LENGTH F : CCC CCC GGC GGC CGC TCA TGG CCG GCG AAG GA
		Reverse	DELETION MID R : ATC CCA TTC ACG TAG TGG CAA AAT TTG CAC
		Forward	DELETION MID F : GTG CAA ATT TTG CCA CTA CGT GAA TGG GAT
		Reverse	FULL LENGTH R : CCC CCC GCT CGA GTT ATA AGC AAA GTA TTA
Crbn-△C	Cloning into pcDNA3.0-HA vector	Forward	CCC CCC GGC GGC CGC TCA TGG CCG GCG AAG GA
		Reverse	CCC CCC GCT CGA GTT ATG GCA AAA TTT GCA C
Crbn-△N	Cloning into pEBB-Triple HA vector	Forward	CCC CCC GGC GGC CGC TCC TAC GTG AAT GGG A
		Reverse	CCC CCC GCT CGA GTT ATA AGC AAA GTA TTA
Crbn-Lon	Cloning into pEBB-Triple HA vector	Forward	CCC CCC GGC GGC CGC TCA TTC CAG TTC TTC CT
		Reverse	CCC CCC GCT CGA GTT TGT TCA TGA TGT CT
Orai1-K80A	Site-mutation K80A AAG(K)→GCG (A)	Forward	GTC CTG GCG CGC GCT CTA CTT AAG CCG CGC CAA
		Reverse	TTA AGT AGA GCG CGC GCC AGG ACA GCG CCT GCA
Orai1-K87A	Site-mutation K87A AAG(K)→GCG(A)	Forward	AAG CCG CGC CGC GCT CAA AGC TTC CAG CCG GAC
		Reverse	AAG CTT TGA GCG CGG CGC GGC TTA AGT AGA GCT
Orai1-K89A	Site-mutation K89A AAA(K)→GCA(A)	Forward	CGC CAA GCT CGC AGC TTC CAG CCG GAC CTC GGC C
		Reverse	GGC TGG AAG CTG CGA GCT TGG CGC GGC TTA AGT
Orai1-K163A	Site-mutation K163A AAA(K)→GCA(A)	Forward	CAA CTC GGT CGC AGA GTC ACC CCA CGA GCG CAT
		Reverse	GGG GTG ACT CTG CGA CCG AGT TGA GGT TGT GGA
Orai1-K268A	Site-mutation K268A AAG(K)→GCG(A)	Forward	GGT CAG CCA TGC GAC GGA CCG GCA GTT CCA GGA
		Reverse	GCC GGT CCG TCG CAT GGC TGA CCA GGG AAC GGT
Crbn	Cloning into pEBB-GFP vector	Forward	ATT GCT AGC ATG GCC GGC GAA GGA GAT C
		Reverse	ATT GGT ACC TAA GCA AAG TAT TAC TTT GTC

Stim1	Cloning into pEBB vector	Forward	AAT ACT AGT ATG GAT GTG TGC GCC CGT
		Reverse	AAT GTT AAC CTA CTT CTT AAG AGG CTT CTT AAA AAT
GS (Glutamine synthetase)	Cloning into pEBB-Flag vector	Forward	ATT ACT AGT ATG GCC ACC TCA GCA AGT TCC
		Reverse	ATT GTC AAC GTT CTT GTA TTG GAA GGG TTC
Human Orai1	Cloning into pEBB-Flag vector	Forward	ATT GCT AGC ATG CAT CCG GAG CCC GCC
		Reverse	ATT GGT ACC GGC ATA GTG GCT GCC GGG
Human Orai1β	Cloning into pEBB-Flag vector	Forward	ATT GCT AGC ATG AGC CTC AAC GAG CAC TCC ATG
		Reverse	ATT GGT ACC GGC ATA GTG GCT GCC GGG
